# Detecting spiritual pain in electronic medical records using large language models

**DOI:** 10.1007/s00520-026-11416-0

**Published:** 2026-10-29

**Authors:** Kento Masukawa, Momoe Meguro, Erika Aihara, Miku Ishida, Uta Kondo, Ryusuke Inoue, Masaharu Nakayama, Mitsunori Miyashita

**Affiliations:** 1 Department of Palliative Nursing, Health Sciences, Tohoku University Graduate School of Medicine, Sendai, Miyagi Japan; 2 Medical Information Technology Center, Tohoku University Hospital, Sendai, Miyagi Japan; 3 Department of Medical Informatics, Tohoku University Graduate School of Medicine, Sendai, Miyagi Japan

**Keywords:** Large language models, Spiritual pain, Natural language processing, Electronic medical records

## Abstract

**Purpose:**

Spiritual pain is one dimension of total pain in terminally ill patients with cancer. Reducing the burden of assessing spiritual pain in electronic medical records is essential for improving the quality of spiritual care. Large language models may offer a solution. This study explored whether large language models can be used to develop an automatic system for accurately identifying spiritual pain in patients with terminal cancer.

**Methods:**

We used unstructured nursing records from patients with cancer who died in an inpatient palliative care unit at a university hospital in Japan between July and December 2024. A total of 500 records generated within 3 weeks before patients’ death were randomly selected for analysis. Records were labeled for the presence of spiritual pain. Gemini-2.5-flash and Microsoft Copilot, accessible within the Tohoku University network environment, were utilized. Prompts included definitions of spiritual pain and example sentences.

**Results:**

The analyzed records were derived from 109 patients with cancer (57 (52.3%) male; mean age, 70.9 years). Manual review identified spiritual pain in 56 records (11.2%). Sensitivity for Gemini and Copilot was 1.00; specificity was 0.75 and 0.61; positive predictive value was 0.34 and 0.25; and negative predictive value was 1.00 and 1.00, respectively. Error analysis of false positives indicated that the models frequently misclassified decreased physical functions and delirium as spiritual pain.

**Conclusion:**

This study demonstrates that large language models can identify spiritual pain with performance comparable to, or exceeding, that of previous studies utilizing classical natural language processing methods.

**Supplementary information:**

The online version contains supplementary material available at https://doi.org/10.1007/s00520-026-11416-0.

## Introduction

Spiritual pain is one dimension of total pain in terminally ill patients with cancer [1]. The World Health Organization proposed that the distress of terminally ill patients should be understood as total pain, encompassing physical symptoms, psychological symptoms, social distress, and spiritual pain [2]. Spiritual pain has been defined as “a pain deep in your soul (being) that is not physical [3]” or as “pain caused by extinction of the being and the meaning of self [4]*.*” Many patients with cancer experience spiritual pain, and spiritual care needs are high among patients and their family caregivers [5–7]. Spiritual pain significantly reduces the quality of life in terminally ill patients with cancer [8, 9]. Therefore, healthcare providers and researchers in palliative care need to assess spiritual pain alongside physical and psychological symptoms [10, 11].

Clinical records in electronic medical records can be valuable for assessing spiritual pain [12]. These records contain narrative data derived from daily conversations between patients and healthcare providers, often including information about spiritual pain [13]. However, manually reviewing such a large volume of narrative records and assessing spiritual pain is time-consuming and highly burdensome for healthcare providers. This workload can lead to overlooking or misunderstanding spiritual pain in terminally ill patients with cancer. To address this challenge, some studies have developed assessment support systems using traditional natural language processing and machine learning to detect the spiritual pain experienced by terminally ill patients with cancer and their family caregivers within electronic medical records [13, 14]. Improving the performance of these systems could lead to a clinical decision support system that enhances the quality of spiritual care for terminally ill patients with cancer [13, 14].

Utilizing large language models may be one approach for further improving the performance of models that detect spiritual pain. These artificial intelligence (AI) models generate responses based on user-entered text (*prompts*). This technology has also been applied in the medical field, with several studies using it to identify specific information from large text datasets contained in electronic medical records [15, 16]. For example, recent studies have demonstrated that existing large language models can identify records related to advanced care planning from electronic medical records as accurately as traditional natural language processing methods, even without a large amount of training data for AI development [17, 18]. Another study showed that large language models can automatically extract heart failure symptoms from electronic medical records with high performance using only simple prompts [19]. Thus, utilizing large language models could potentially enable the development of a highly accurate system for automatically identifying spiritual pain from electronic medical records without requiring large datasets. However, no studies have evaluated the extent to which existing large language models can identify spiritual pain in terminally ill patients with cancer from unstructured electronic medical records.

Therefore, this study aimed to explore whether using large language models can be useful for developing an accurate automatic system for identifying spiritual pain in patients with terminal cancer.

## Methods

### Study design

This study was part of a retrospective cohort study approved by the Tohoku University Institutional Review Board (Approval No. 2024-1-1006).

### Study population and data source

The study included patients who died in an inpatient palliative care unit of Tohoku University Hospital in Japan between July and December 2024. We abstracted and de-identified free-text nursing records (Subjective–Objective–Assessment–Plan notes) from the electronic medical record system at Tohoku University Hospital within 3 weeks before each patient’s death (*n* = 6858).

To reduce annotation burden, we filtered the nursing records using search terms related to spiritual pain. We applied 20 core search terms identified in a previous study (Appendix A) and expanded these terms using distributed representations of each term [13]. Using exact string matching, we identified 1808 nursing records containing 558 search terms. From these, 500 nursing records were randomly selected for analysis. The mean record length ± standard deviation (SD) was 349.9 ± 143.0 characters.

### Spiritual pain labels

Following the detection guideline used in a previous study (Appendix B) [13], each record was labeled for the presence of spiritual pain (0: absence; 1: presence). Two researchers independently labeled each record according to the guidelines, and any disagreements were resolved through discussion. Subsequent to independent labeling, a consensus meeting was held to review all cases of disagreement. Discrepancies were meticulously examined case-by-case by referring to the guidelines until consensus was achieved. Should consensus fail to emerge from discussion, a third senior researcher in palliative care was available to review and make the final decision; however, all discrepancies between the two primary raters in this study were successfully resolved. The guideline was based on the *spiritual pain assessment sheet (SpiPas)* developed in Japan for nurses [20, 21]. SpiPas defines spiritual pain as pain caused by the extinction of being and meaning of self. Spiritual pain involves three dimensions of human existence: being grounded in temporality, being in relationships, and being autonomous. Records that exclusively documented “spiritual pain present” without detailing a particular subdomain were also labeled as positive for spiritual pain (categorized as “Others”). Prior to consensus discussion, inter-rater reliability was evaluated using Cohen’s kappa coefficients, demonstrating moderate to practically perfect agreement across categories. The kappa values recorded were 0.85 for autonomy, 0.87 for temporality, 0.74 for relationships, and 0.72 for others.

### Large language models and prompts

We used two large language models (Gemini-2.5-flash and Microsoft Copilot) that are accessible within the Tohoku University network environment. Prompts were constructed and executed in Japanese utilizing a few-shot prompting strategy to match the language of the electronic medical records. This few-shot prompting strategy provided the models with operational definitions of spiritual pain along with concrete sentence examples. Table 1 illustrates the prompt employed in this study. The prompt content comprised: (1) directives for instructions, (2) operational definitions of spiritual pain and its subdomains based on SpiPas, and (3) the designated nursing record.

**Table 1:** Content of the prompt for large language models

### Instruction
The following text is a medical record from an electronic medical record system. Read the text and, based on the definitions provided, assess whether the patient is experiencing spiritual pain. Please also indicate which aspect of spiritual pain the patient may be experiencing.
###Definition
(See Appendix A)
###Medical records
. . . . . .

### Performance evaluation

Model performance was evaluated using sensitivity, specificity, positive predictive value, and negative predictive value. Additionally, we conducted an error analysis of false-positive cases. The large language models output reasons for detections, indicating the presence of spiritual pain. Two researchers independently conducted a qualitative analysis of cases and categorized error types based on similarities. Discrepancies in categorization were discussed and resolved between researchers. The final list of categories was reviewed by all researchers and consolidated. All analyses were conducted using Python (version 3.14) and R (version 4.5.2).

## Results

### Patient characteristics

 We analyzed data from 109 patients with cancer. The patients’ characteristics are summarized in Table 2. Their mean age ± SD was 70.9 ± 11.1 years, and 57 patients (52.3%) were male. The most frequent primary cancer site was head and neck (*n* = 14), followed by pancreatic cancer (*n* = 13).

**Table 2:** Patients’ characteristics

	*n*	%
Age (mean ± SD)	(70.9 ±	11.1)
Sex		
Male	57	52.3
Female	52	47.7
Primary cancer site		
Brain	5	4.6
Head and neck	14	12.8
Breast	7	6.4
Lung	9	8.3
Gastric and esophageal	12	11.0
Hepatobiliary and pancreatic	20	18.3
Colon and rectum	12	11.0
Urology	11	10.1
Uterus and ovary	5	4.6
Blood	5	4.6
Others	9	8.3

*SD* standard deviation

### Spiritual pain labeling outcomes

Table 3 presents the number of records labeled as indicating the presence of spiritual pain. In the manual chart review, 56 records (11.2%) were identified as indicating the presence of spiritual pain.

**Table 3:** Number of records labeled for the presence of spiritual pain

	*n*	%
*Manual chart review*		
Any spiritual pain	56	11.2
Autonomy	23	4.6
Relationships	18	3.6
Temporality	21	4.2
Others	5	1.0
*Evaluation by Gemini*		
Any spiritual pain	167	33.4
Autonomy	103	20.6
Relationships	30	6.0
Temporality	64	12.8
Others	11	2.2
*Evaluation by Copilot*		
Any spiritual pain	228	45.6
Autonomy	153	30.6
Relationships	36	7.2
Temporality	52	10.4
Others	13	2.6

### Model performance

Table 4 outlines model performance. For the detection of any spiritual pain, the sensitivity for Gemini and Copilot was 1.00 (95% confidence interval, 0.91–1.00) and 1.00 (0.91–1.00), respectively; specificity was 0.75 (0.71–0.79) and 0.61 (0.57–0.66); positive predictive value was 0.34 (0.26–0.41) and 0.25 (0.19–0.31); negative predictive value was 1.00 (0.98–1.00) and 1.00 (0.98–1.00); and accuracy was 0.78 (0.74–0.81) and 0.66 (0.61–0.70). For detection of spiritual pain subdomains, sensitivity ranged from 0.74 to 0.91; specificity, from 0.72 to 0.99; positive predictive value, from 0.11 to 0.57; negative predictive value, from 0.98 to 1.00; and accuracy, from 0.71 to 0.98.

**Table 4:** Performance of large language models for detecting spiritual pain

	Sensitivity (95% CI)	Specificity (95% CI)	PPV (95% CI)	NPV (95% CI)	Accuracy (95% CI)
Any spiritual pain					
*Gemini*	1.00 (0.91–1.00)	0.75 (0.71–0.79)	0.34 (0.26–0.41)	1.00 (0.98–1.00)	0.78 (0.74–0.81)
*Copilot*	1.00 (0.91–1.00)	0.61 (0.57–0.66)	0.25 (0.19–0.31)	1.00 (0.98–1.00)	0.66 (0.61–0.70)
Autonomy					
*Gemini*	0.87 (0.66–0.97)	0.83 (0.79–0.86)	0.19 (0.12–0.28)	0.99 (0.98–1.00)	0.83 (0.79–0.86)
*Copilot*	0.74 (0.52–0.89)	0.72 (0.67–0.76)	0.11 (0.07–0.17)	0.98 (0.96–0.99)	0.71 (0.67–0.76)
Relationships					
*Gemini*	0.94 (0.72–1.00)	0.97 (0.95–0.99)	0.57 (0.37–0.75)	1.00 (0.99–1.00)	0.97 (0.95–0.99)
*Copilot*	0.78 (0.52–0.94)	0.95 (0.92–0.97)	0.39 (0.23–0.57)	0.99 (0.98–1.00)	0.94 (0.92–0.96)
Temporality					
*Gemini*	0.87 (0.76–1.00)	0.91 (0.88–0.93)	0.31 (0.20–0.44)	1.00 (0.99–1.00)	0.91 (0.88–0.93)
*Copilot*	0.91 (0.70–0.99)	0.93 (0.91–0.95)	0.37 (0.24–0.51)	1.00 (0.98–1.00)	0.93 (0.90–0.95)
Others					
*Gemini*	0.80 (0.28–1.00)	0.99 (0.97–0.99)	0.36 (0.11–0.69)	1.00 (0.99–1.00)	0.98 (0.97–0.99)
*Copilot*	0.80 (0.28–1.00)	0.98 (0.97–0.99)	0.31 (0.09–0.61)	1.00 (0.99–1.00)	0.98 (0.96–0.99)

*CI* confidence interval, *PPV* positive predictive value, *NPV* negative predictive value

### Error analysis

We conducted an error analysis of Gemini’s detections. Table 5 presents the results of false-positive error analysis (*N* = 111). Seven error types were identified: (1) interpreting deteriorating physical function as spiritual pain (*n* = 44); (2) interpreting delirium as spiritual pain (*n* = 28); (3) overinterpretation of the patient’s words (*n* = 15); (4) interpreting physical symptoms as spiritual pain (*n* = 15); (5) interpreting perception of prognosis as spiritual pain (*n* = 5); (6) interpreting psychological symptoms as spiritual pain (*n* = 2); and (7) others (*n* = 2). Representative cases of valid true positive identifications made by the models across the SpiPas subdomains are detailed in Appendix C.

**Table 5:** Examples of false-positive cases (*N* = 111)

Error type	Examples of records
1. Interpreting deteriorating physical function as spiritual pain (*n* = 44)	• *S: I want to go to the restroom* *O: The patient has pressed the nurse call button. He requests wheelchair assistance to the restroom, but due to significant lower limb edema, he is unable to sit up independently* • *S: I don’t feel like eating. I won’t be having dinner* *O: Patient has no appetite and is unable to eat dinner* *A: Significant loss of appetite. The patient’s overall condition is deteriorating*
2. Interpreting delirium as spiritual pain (*n* = 28)	• *O: The patient is restless, repeatedly sitting up and lying back down in bed. A: The delirium is becoming more severe* *S: I don’t know what to do* *O: Even after administering Serenace, the patient remains confused, cycling between drowsiness and wakefulness* *A: The patient is deemed to be in terminal delirium and experiencing significant distress*
3. Overinterpretation of the patient’s words (*n* = 15)	• *S: I just want to sleep, but I still must take my medicine. Being woken up is painful for me too* • *S: My friend is coming over this afternoon, but I’m feeling restless because I don’t know what time they’ll arrive*
4. Interpreting physical symptoms as spiritual pain (*n* = 15)	• *S: I feel short of breath when I move* *O: Patient was asleep but complained of dyspnea. SPO2: 78–79% under 6 L/min oxygen *via* mask. RR 20 breaths/min. No phlegm* *A: Complaint of dyspnea upon awakening. Midazolam was administered to ensure continued sleep*
5. Interpreting perception of prognosis as spiritual pain (*n* = 5)	• *S: I’ve got about a week left to live…* *A: The patient is aware of their poor condition and seems to understand their prognosis*
6. Interpreting psychological symptoms as spiritual pain (*n* = 2)	• *S: Feeling emotionally distressed* *O: No physical pain, but reports feeling emotionally distressed* *A: Experiencing psychological distress and appears to desire rest*
7. Others (*n* = 2)	• The medical record contains only the notation “spiritual pain.” • *S: Thank you* *O: No redness or swelling around the left auricle (self-inflicted wound)* *A: Pain control is good. Mentally calm*

## Discussion

To the best of our knowledge, this is the first study to use large language models and evaluate their performance in detecting spiritual pain from unstructured text data in electronic medical records. This study demonstrates the feasibility and potential utility of large language models as high-sensitivity preliminary screening tools for spiritual pain in electronic medical records. Additionally, performance may be improved by incorporating prompts that specifically focus on spiritual pain and distinguish it from other symptoms.

This study showed that large language models can detect spiritual pain from nursing records with high sensitivity. In a previous study, models employing classical natural language processing were used to detect spiritual pain in terminally ill patients with cancer, achieving a sensitivity of 0.82, specificity of 0.84, positive predictive value of 0.28, and negative predictive value of 0.98 [13]. The current study demonstrates that contemporary large language models can achieve comparable performance to that of previous approaches. Several studies have also reported that large language models can capture specific clinical information from unstructured text data in electronic medical records with high performance, comparable to that of traditional methods [17, 19, 22]. Furthermore, this study highlights the utility of pretrained general-purpose large language models, which are not specifically trained on clinical medical records, enabling the development of systems for detecting spiritual pain from electronic medical records without requiring large amounts of training data.

Error analysis of false positives revealed that the large language model and prompt employed in this study tend to misclassify deteriorating physical function or delirium as spiritual pain. The recurrent misclassification reflects the inherent clinical intricacy of end-of-life care. Deteriorating physical function and delirium can act as triggers for spiritual pain [20]. This establishes a notable overlap in clinical frameworks and nursing documentation, complicating differentiation even for human evaluators. The false positives may stem not only from the misclassification within the model but may also be influenced by the ambiguity associated with physical and spiritual distress in actual clinical nursing records. Clarifying these distinctions within the prompts could enhance the performance of spiritual pain identification. Additionally, incorporating prompts that discourage judgments based on ambiguous expressions and require adherence to strict definitions may further improve performance [23]. Moreover, the Chain-of-Thought (CoT) prompting method may also be useful. The CoT is a technique that guides the reasoning process step by step toward an output [24]. For example, a hierarchical approach that first uses the prompt from this study to assess spiritual pain and then applies a second prompt to distinguish other symptoms and spiritual pain may be effective. Future research should examine whether these prompt design strategies can improve the performance of spiritual pain detection.

Despite these challenges, the current prompt can serve as a high-sensitivity preliminary screening method to identify patients at high risk of spiritual pain and facilitate referral for appropriate support. The positive predictive values were suboptimal, suggesting that the current approach may generate a considerable number of false positives that could increase clinicians’ burden (e.g., alert fatigue). Therefore, future investigations should prioritize establishing two-stage screening processes involving clinician verification to improve positive predictive value and reduce clinical burden. Despite the fact that the method used in this study is not yet sufficient and could not be used as a standalone diagnostic system, it demonstrates initial feasibility and possible effectiveness as a screening support tool to help healthcare professionals in identifying patients’ spiritual care needs.

Integrating a large language model into electronic medical records can contribute to improving the quality of palliative care, including spiritual care. Several studies have shown that healthcare staff currently face challenges in adequately providing spiritual care to patients, and that education on spiritual pain and spiritual care remains insufficient [25–27]. Moreover, the anticipated increase in patients requiring palliative care makes it essential for non-specialists to address spiritual pain as well [28]. Thus, developing a highlighting system within electronic medical records to indicate the presence of spiritual pain would enable even non-specialists to recognize it and incorporate it into care plans. Furthermore, future research should not only focus on detecting spiritual pain using large language models but also validate the clinical utility of spiritual care recommendations generated by these models [14]. If large language models can generate clinically valid recommendations for spiritual care, they could enable healthcare providers to deliver more timely and high-quality support, potentially enhancing palliative care quality and contributing to a new educational approach.

### Limitations

This study has some limitations. First, we analyzed data from a single university hospital in Japan, which limits the generalizability of the findings. Further studies should evaluate performance using datasets from other institutions and in other languages. Second, this study used only two large language models that were available on-site due to personal information protection requirements; other models may achieve different performance levels. Third, the sample size was relatively small. A total of 500 nursing records were collected from 109 patients, and multiple records from the same individuals were included. This continuous sampling from the same patients may have limited the overall diversity of nursing documentation styles, potentially leading to model performance overestimation. Future studies should validate these models in larger patient cohorts. Fourth, the dataset was first subjected to pre-filtering using search terms pertaining to spiritual pain prior to randomly sampling 500 records. This pre-filtering approach may have introduced selection bias and augmented the dataset with relevant narrative contexts. Thus, the performances in this study may differ from or be overestimated, compared with performance in real-world clinical datasets. Future studies should therefore validate these models on completely unfiltered electronic medical records.

## Conclusion

In this study, we evaluated the performance of large language models in detecting spiritual pain from unstructured text data in electronic medical records among terminally ill patients with cancer. This study demonstrates the feasibility and prospective usefulness of large language models as high-sensitivity preliminary screening tools for spiritual pain in electronic medical records. Given the low positive predictive value and risk of clinician alert fatigue, further research is required to refine prompt strategies and develop two-tiered screening processes with clinician verification before these models can be reliably integrated into routine clinical practice.

## Supplementary information

Below is the link to the electronic supplementary material.

Supplementary file1 (DOCX 29 kb)

## Data Availability

The data used in this study are available on request from the corresponding author. The data are not publicly available owing to privacy and ethical restrictions.
