# Supplementary material for: Detecting spiritual pain in electronic medical records using large language models
Source: Support Care Cancer. 2026 Oct 29;34(11):1167. doi: 10.1007/s00520-026-11416-0 (PMC13719773; doi:10.1007/s00520-026-11416-0)
Supplement: Supplementary file 1 [file 520_2026_11416_MOESM1_ESM.docx]

**Appendix A. Core search terms**

Spiritual pain

Spiritual

Loneliness

View of life and death

Hardship

Sorry

God

Why

Religious

Tear

Hardship

Miserable

Life

Regretful

Death

Incorporeal

*Hotoke*

Purpose of life

Inheritance

Guilt

Annoyance

**Appendix B. Abstraction guidelines for assessing spiritual pain**

Spiritual pain was defined in accordance with the Spiritual Pain Assessment Sheet (SpiPas). The SpiPas was based on findings from observational studies conducted with terminally ill patients, families, and spiritual care professionals in Japan. It defines spiritual pain as suffering caused by the extinction of one’s being and loss of meaning of the self. Spiritual pain consists of three dimensions: relationships, autonomy, and temporality. Clinical notes were labeled when they referred to any of these dimensions or when clinical staff explicitly assessed that a patient was experiencing spiritual pain.

*Element 1: Relationships*

The relationships dimension consists of the following four factors: concerns about family, isolation, burden to others, and transcendental being or force.

A) Concerns about family
   - “I’m worried about my wife after I’m gone.”
B) Isolation
   - “No one understands me.”
   - “When my family leaves, I feel alone.”
C) Burden to others
   - “I’m sorry to bother you.”
   - “I can’t do anything without someone.”
D) Transcendental being or force
   - “Neither God nor Buddha saved me…”
   - “We can’t do anything about the forces of nature.”

*Element 2: Autonomy*

The autonomy dimension consists of the following five factors: independence; loss of control over the future; loss of purpose and enjoyment; loss of the sense of self; and change in body image.

Examples include:

A) Independence
   - “I am unable to go to the bathroom. It’s miserable.”
   - “It’s hard for me not be able to move.”
B) Loss of control over the future
   - “I’m afraid that I don’t know what will happen in the future.”
C) Loss of purpose and enjoyment
   - “I cannot do anything. It’s not worth living.”
   - “It’s hard for me to fulfill my role as a mother.”
D) Loss of sense of self
   - “I can’t do anything to make my life worth living.”
E) Change in body image
   - “I don’t want anyone to see me this way.”
   - “Don’t see me depressed.”

*Elements 3: Temporality*

The temporality dimension consists of the following five factors: regret, hopelessness, anxiety over the prospect of death, worry about putting one’s affairs in order, and the absurdity of life.

A) Regret
   - “I’m sad that I won’t be able to watch my children grow up.”
   - “I won’t see my grandchildren.”
B) Hopelessness
   - “There is nothing fun to do.”
   - “Doctor, please kill me quickly.”
C) Anxiety over the prospect of death
   - “I’m afraid of dying.”
   - “What will happen if I die?”
D) Worry about putting one’s affairs in order
   - “I worry about work that remains undone.”
E) Absurdity of life
   - “Why do I have to suffer so much?”
   - “The reason I’m suffering so much is that I’m being punished.”

**Appendix C. Examples of true-positive cases**

| **Spiritual pain sub-domain** | **Example of records** |
| --- | --- |
| Relationships | *S: I feel bad about having to ask others for help with things like going to the bathroom.*  *O: While in bed bath, she quietly speaks the above with tears in her eyes.*  *A: This patient is experiencing emotional distress due to difficulty moving.* |
| Autonomy | *S: (Unable to stand) I can’t move at all—what should I do?*  *O: The patient expressed a desire to transfer to a portable toilet; two nurses assisted the patient into a sitting position, but the patient was unable to stand and returned to bed.* |
| Temporality | *S: I want to end my life. How can I do that? Everything is so painful.*  *O: Although the patient is not experiencing physical pain due to medication adjustments, they are experiencing psychological distress.*  *A: The patient has expressed suicidal ideation. We provide support to help alleviate the patient’s suffering.* |
| Others | *A: Experiencing spiritual pain* |
